# Dietary Corn Starch Levels Regulated Insulin-Mediated Glycemic Responses and Glucose Homeostasis in Swimming Crab (*Portunus trituberculatus*)

**DOI:** 10.1155/2022/2355274

**Published:** 2022-09-06

**Authors:** Xiangsheng Zhang, Chaokai Huang, Yuhang Yang, Xiangkai Li, Chen Guo, Zheng Yang, Shichao Xie, Jiaxiang Luo, Tingting Zhu, Wenli Zhao, Min Jin, Qicun Zhou

**Affiliations:** Laboratory of Fish and Shellfish Nutrition, School of Marine Sciences, Ningbo University, Ningbo 315211, China

## Abstract

Carbohydrate is the cheapest source of energy among the three major nutrient groups, an appropriate amount of carbohydrates can reduce feed cost and improve growth performance, but carnivorous aquatic animals cannot effectively utilize carbohydrates. The objectives of the present study are aimed at exploring the effects of dietary corn starch levels on glucose loading capacity, insulin-mediated glycemic responses, and glucose homeostasis for *Portunus trituberculatus*. After two weeks of feeding trial, swimming crabs were starved and sampled at 0, 1, 2, 3, 4, 5, 6, 12, and 24 hours, respectively. The results indicated that crabs fed diet with 0% corn starch exhibited lower glucose concentration in hemolymph than those fed with the other diets, and glucose concentration in hemolymph remained low with the extension of sampling time. The glucose concentration in hemolymph of crabs fed with 6% and 12% corn starch diets reached the peak after 2 hours of feeding; however, the glucose concentration in hemolymph of crabs fed with 24% corn starch attained the highest value after 3 hours of feeding, and the hyperglycemia lasted for 3 hours and decreased rapidly after 6 hours of feeding. Enzyme activities in hemolymph related to glucose metabolism such as pyruvate kinase (PK), glucokinase (GK), and phosphoenolpyruvate carboxykinase (PEPCK) were significantly influenced by dietary corn starch levels and sampling time. Glycogen content in hepatopancreas of crabs fed with 6% and 12% corn starch first increased and then decreased; however, the glycogen content in hepatopancreas of crabs fed with 24% corn starch significantly increased with the prolongation of feeding time. In the 24% corn starch diet, insulin-like peptide (ILP) in hemolymph reached a peak after 1 hour of feeding and then significantly decreased, whereas crustacean hyperglycemia hormone (CHH) was not significantly influenced by dietary corn starch levels and sampling time. ATP content in hepatopancreas peaked at 1 h after feeding and then decreased significantly in different corn starch feeding groups, while the opposite trend was observed in NADH. The activities of mitochondrial respiratory chain complexes I, II, III, and V of crabs fed with different corn starch diets significantly increased first and then decreased. In addition, relative expressions of genes related to glycolysis, gluconeogenesis, glucose transport, glycogen synthesis, insulin signaling pathway, and energy metabolism were significantly affected by dietary corn starch levels and sampling time. In conclusion, the results of the present study reveal glucose metabolic responses were regulated by different corn starch levels at different time points and play an important role in clearing glucose through increased activity of insulin, glycolysis, and glycogenesis, along with gluconeogenesis suppression.

## 1. Introduction

Carbohydrate is not the main source of energy for aquatic animals and the main components of aquafeeds [1]; however, it has a lot of advantages such as its low cost, most widely available, and most abundant of the three energy sources [2]. With the rapid development of aquafeed worldwide, especially in China, the prices of high-quality protein and lipid sources such as fish meal and fish oil have rapidly risen. Under this severe situation, it is the general trend to add an appropriate proportion of carbohydrates to aquafeeds. Appropriate carbohydrate supplementation in aquafeed can not only reduce the cost of feed production, alleviate the contradiction of shortage of feed resources, but also even improve growth and feed utilization capacity of aquatic animals [3–5]. Although the addition of carbohydrates to aquafeed has many advantages, many studies indicated that dietary carbohydrates caused glucose intolerance of aquatic animals, especially carnivorous aquatic animals [1]. Previous studies have demonstrated that it takes 5-8 hours to clear their glucose load for herbivorous or omnivorous fish, such as grass carp (*Ctenopharyngodon idellus*) [6], tilapia (*Oreochromis niloticus*) [7], gibel carp (*Carassius gibelio*) [8]. However, the high blood glucose of carnivorous fish lasts for more than 12 hours after being loaded with glucose or consuming high-carbohydrate aquafeed, such as rainbow trout (*Oncorhynchus mykiss*) [9], Japanese flounder (*Paralichthys olivaceus*) [10], and grouper (*Epinephelus coioides*) [11]. Glucose intolerance, a condition in which the glucose load exceeds the body's ability to clear it, resulted in persistent high blood glucose. Generally, it is judged by a glucose tolerance test; that is, after injecting or taking a high dose of glucose or carbohydrate to the body and observing whether the body has persistent hyperglycemia symptoms, if the body's blood glucose does not return to the basic level within 1-2 hours, it can be judged as impaired glucose tolerance [12]. Only mammals can reach blood basal glucose levels within 1-2.5 hours after a glucose load [13], and glucose intolerance can cause persistent high blood glucose that not only affects normal growth but also harms body health, such as liver injury [4, 14, 15]. The swimming crab owns an open circulatory system, and the injection will have a very high fatality rate, so this experiment simulates the glucose load effect brought by the tolerance test through the feeding test.

The swimming crab, *Portunus trituberculatus*, is widely distributed in the coastal seawater of China, South Korea, and Japan and has become one of the most important economically farmed species [16]. Due to its rapid growth, delicious meat, and balanced nutrition, it has huge market value and potential. Since the 1990s, the breeding industry of swimming crabs has developed rapidly in the coastal areas in China, and in recent years, the production of swimming crabs has stabilized [17]. According to the 2021 China Fishery Statistical Yearbook, the output of artificially cultured swimming crabs will reach 100,895 tons in 2020 [17]. Therefore, to improve the feed formula of swimming crabs, reduce the cost pressure, and make the swimming crabs grow healthily and rapidly, it is very necessary to find out the tolerance of swimming crabs to carbohydrate substances. An 8-week feeding trial has been carried out to determine the appropriate supplemental level of corn starch in feed [18]. The short-term feeding experiment is to further explore the effects of dietary corn starch levels on glucose tolerance, enzyme activities related to carbohydrate metabolism, hormones, gene expression, and mitochondrial homeostasis of the swimming crab.

## 2. Materials and Methods

### 2.1. Ethics Statement

All experimental procedures were performed in strict accordance with the standard operation procedures (SOPs) of the *Guide for Use of Experimental Animals* of Ningbo University. The experimental protocol and procedures were approved by the Institutional Animal Care and Use Committee of Ningbo University (NBU-2022-R01).

### 2.2. Experimental Design and Feed Preparation

Four isoproteic (43% crude protein) and isolipidic (6% crude lipid) diets were formulated to contain 0, 6, 12, and 24% corn starch, and the actual contents of nitrogen-free extract (NFE) were 2.14, 7.43, 14.00, and 25.30%, respectively (Table 1). All ingredients were ground into fine powder by ultrafine grinder, and the vitamin, mineral mixture and other raw materials (less than 100 g) were mixed firstly, then added into the large amount of raw materials. Cold-extruded pellets were produced (F-26, Machine Factory of South China University of Technology) with pellet strands cut into uniform sizes (4 mm diameter pellets) (G-250, Machine Factory of South China University of Technology). Pellets were steamed for 30 min at 90°C and finally air-dried to approximately 100.0 g kg^−1^ moisture. The dried diets were sealed in vacuum-packed bags and stored at -20°C until used.

### 2.3. Feeding Trial and Experimental Conditions

Swimming crabs were purchased from a local crab breeding farm (Xiangshan, Ningbo, China). Feeding trial was conducted in Ningbo Xiangshan Harbor Aquatic Seed Co. Ltd. Healthy and similar-sized juvenile swimming crabs were acclimated for 2 weeks and fed with a commercial diet (45% dietary protein and 8% crude lipid, Ningbo Tech-Bank Feed Co., Ltd., Ningbo, China) before the start of the feeding trial. The initial average weight of 240 swimming crab juveniles was 50.87 ± 0.5 g and randomly sorted into 240 individual rectangle plastic baskets (35 × 30 × 35 cm) in three cement pools (6.8 × 3.8 × 1.7 m). Each diet was randomly assigned to three replicates, and each replicate had 20 plastic baskets that were supported with a foam frame so that it does not sink to the bottom. All crabs were fed once at 18:00, and the moulting crab was not fed. Feces, uneaten feed, and crab shells were removed in the morning after the feeding, and about 30% of seawater in the cement pool was daily exchanged to maintain seawater quality. Furthermore, the amounts of uneaten complete pellets (crushed residue feed is not counted) and dead crabs were recorded, weighed, and calculated. In order to continuously supply oxygen, 12 air pipes connected to air stones were placed in the cement pool for continuous aeration. During the experimental period, the temperature of the cement pool was 27-30.5°C, the salinity was 22.5-25.5 g L^−1^, pH was 7.3-8.0, and ammonia nitrogen was lower than 0.05 mg L^−1^.

### 2.4. Sample Collection

At the end of the experiment, samples were taken at 0, 1, 2, 3, 4, 5, 6, 12, and 24 hours after feeding, and four crabs were sampled per replicate at each time point. Hemolymph samples from each crab were removed from the pericardial cavity using a 2 mL syringe, sorted into 1.5 mL Eppendorf tubes overnight at 4°C, and centrifuged at 3500 rpm for 10 min (Eppendorf centrifuge 5810R, Germany). Then, the supernatant was collected and stored at -80°C until analysis of glucose concentration and enzyme activities related to carbohydrate metabolism. The hepatopancreas of four crabs after the hemolymph being taken were drawn and immediately collected into 2 mL Eppendorf tubes and 1.5 mL Eppendorf tubes, 2 mL Eppendorf tubes were frozen in liquid nitrogen and stored at -20°C for glycogen, ATP, NADH, and mitochondrial respiratory chain and other indicators of the determination, and 1.5 mL Eppendorf tube was stored at -80°C for the determination of gene expression.

### 2.5. Enzyme Activity in Hemolymph

The glucose concentration and the activities of pyruvate kinase (PK), glucokinase (GK), phosphofructose kinase (PFK), and phosphoenolpyruvate carboxykinase (PEPCK) in hemolymph were determined using detection kits (Nanjing Jiancheng Bioengineering Institute, China). Glucose concentration was determined by the oxidase method. The enzyme activities related to glucose metabolism were determined by the UV method.

GK was measured using a double-antibody one-step sandwich enzyme-linked immunosorbent assay (ELISA). Specimens, standards, and HRP-labeled detection antibodies were added to precoated GK antibody-coated wells, incubated, and washed thoroughly. The color was developed with the substrate TMB. TMB becomes blue under the catalysis of peroxidase and finally yellow under the action of acid. The shade of color is positively correlated with GK in the sample. Measure the absorbance (OD value) at a wavelength of 450 nm with a microplate reader to calculate the sample concentration.

PK can catalyze PEP to produce pyruvate in the presence of ADP, which is then converted into lactate by LDH and NADH into NAD^+^. PFK catalyzes fructose-6-phosphate and ATP to generate fructose-1,6-bisphosphate and ADP. PK, and LDH further catalyzes NADH to generate NAD^+^. And the rate of decline of NADH is measured at 340 nm to calculate PFK activity. PEPCK catalyzes the reaction of oxaloacetate to generate phosphoenolpyruvate and carbon dioxide, and pyruvate and lactate dehydrogenase further catalyzes NADH to generate NAD^+^, and the reduction rate of NADH is measured at 340 nm to calculate PEPCK activity.

### 2.6. Hormone Content in Hemolymph

The contents of insulin-like peptides (ILP) and crustacean hyperglycemic hormones (CHH) in hemolymph were determined using kits (Jiangsu Kete Biotechnology Co., Ltd., China). Insulin-like peptides and hyperglycemic hormones were also measured by double-antibody one-step sandwich enzyme-linked immunosorbent assay (ELISA). The corresponding antibody can be used.

### 2.7. Energy Homeostasis and Mitochondrial Respiratory Chain Complex in Hepatopancreas

Glycogen, ATP (Nanjing Jiancheng Bioengineering Institute, China), and NADH (Shanghai Biyuntian Biotechnology Co., Ltd., China) were determined using kit assays. The activities of hepatopancreatic mitochondrial respiratory chain complexes I, II, III, and V were also determined by kit assay (Jiangsu Kete Biotechnology Co., Ltd., China).

Glycogen and ATP were determined by colorimetry. Under the action of concentrated sulfuric acid, glycogen can generate aldehyde derivatives and then react with anthrone to generate blue compounds, which are compared with the standard glucose solution treated by the same method for colorimetric comparison. ATP and creatine are catalyzed by creatine kinase to generate phosphocreatine, which is determined by phosphomolybdic acid colorimetry.

NAD^+^ and NADH were determined by WST-8 chromogenic assay. Ethanol is oxidized to acetaldehyde under the action of alcohol dehydrogenase, and NAD^+^ is reduced to NADH. The generated NADH reduces WST-8 to orange-yellow formazan under the action of an electronic coupling reagent, with a maximum absorption peak around 450 nm. The formazan produced in the reaction system is proportional to the total amount of NAD^+^ and NADH in the sample.

Mitochondrial respiratory chain complexes I, II, III, and V were analyzed to use double-antibody one-step sandwich enzyme-linked immunosorbent assay (ELISA) and use corresponding antibodies.

### 2.8. Real-Time Quantitative PCR

RNA extraction and PCR analysis were referred as the method described by Yuan et al. [19]. All specific primers and housekeeping genes were designed by Primer Premier 5.0, synthesized by BGI (Beijing Genomics Research Institute, Shenzhen, China) and verified to be usable (Table 2). In the present study, the expression of genes related to hepatopancreatic carbohydrate metabolism key enzymes, insulin signaling pathway, and transcriptions of mitochondrial function-related genes in swimming crabs was studied. All gene expression data were expressed relative to the expression of the 0% corn starch level diet. The fluorescence data were normalized to *β*-actin and quantified by the 2^−*ΔΔ*Ct^ method [20].

### 2.9. Calculations and Statistical Analysis

Data are presented as the means and standard errors of three replicates (*n* = 3) and analyzed by one-way ANOVA followed by Tukey's multiple-range test. All statistical analyses were conducted using SPSS 23.0 for Windows.

## 3. Results

### 3.1. Glucose Concentrations in Hemolymph

**Figure 1:**
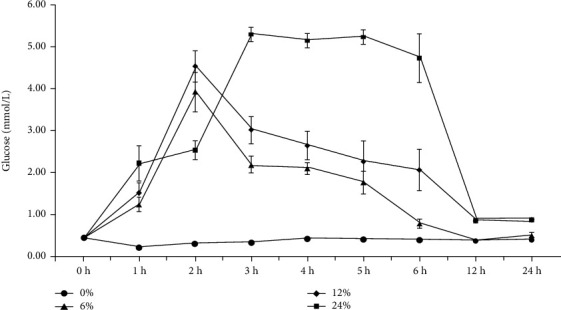
Glucose concentrations in hemolymph of crabs fed with different corn starch levels at different time points (0, 1, 2, 3, 4, 5, 6, 12, and 24 h). Values are expressed as the means ± SE (*n* = 3).

Glucose concentrations in hemolymph of swimming crabs fed with corn starch levels at different time points are presented in Figure 1. The glucose concentration of crabs fed with 0% corn starch diet exhibited a lower level and had no significant change at different time points. Crabs fed diets with 6 and 12% corn starch reached a peak at 2 h after feeding and then decreased to the lowest and remained stable after 12 h. However, crabs fed diet with 24% corn starch, the glucose concentration peaked at 3 hours after feeding and then maintained for 6 h after feeding and then began to decrease significantly, and glucose concentration did not decrease to a minimum and remained stable until 12 h after feeding.

### 3.2. Enzyme Activities Related to Carbohydrate Metabolism in Hemolymph

**Figure 2:**
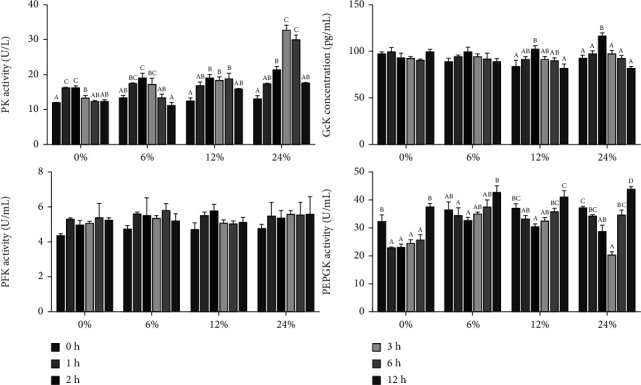
Effects of dietary corn starch levels on hemolymph enzyme activities related to carbohydrate metabolism at different time points (0, 1, 2, 3, 6, and 12 h) for *Portunus trituberculatus.* Values are expressed as the means ± SE (*n* = 3). Means in each bar with different superscript letters are significantly different (*P* < 0.05). PK: pyruvate kinase; GK: glucokinase; PFK: phosphofructokinase; PEPCK: phosphoenolpyruvate carboxykinase.

The effects of different corn starch levels on enzyme activities related to carbohydrate metabolism in hemolymph at different time points are shown in Figure 2. Pyruvate kinase (PK) activity in the hemolymph of crabs fed with different corn starch showed a trend of first increasing and then decreasing with different time points (*P* < 0.05), and the PK activity in hemolymph of crabs fed diet with 0, 6, and 12% corn starch significantly increased at 0-2 h and decreased significantly after 2 h after feeding (*P* < 0.05); however, PK activity in hemolymph of crabs fed diet with 24% corn starch was significantly increased during the ingestion period of 0-3 h and significantly decreased after 3 h of feeding (*P* < 0.05).

In the 0 and 6% corn starch levels, a noticeable change was not found in the glucokinase (GK) activity of swimming crabs (*P* > 0.05), while in the 12 and 24% corn starch diets, GK activity reached a peak at 2 hours after feeding and then significantly decreased (*P* < 0.05). Phosphofructose kinase (PFK) in hemolymph was not significantly influenced by dietary starch levels at different time points (*P* > 0.05). In the 0, 6, 12, and 24% corn starch levels, the activity of phosphoenolpyruvate carboxykinase (PEPCK) in hemolymph decreased first and then increased (*P* < 0.05), and the PEPCK activity decreased significantly during the 0-2 h period of ingestion and began to rebound after 2 h (*P* < 0.05). The PEPCK activity in hemolymph of crabs fed diet with 24% corn starch reached the peak at 3 hours after feeding and significantly increased after 3 h of feeding (*P* < 0.05).

### 3.3. Hormone Concentrations in Hemolymph

**Figure 3:**
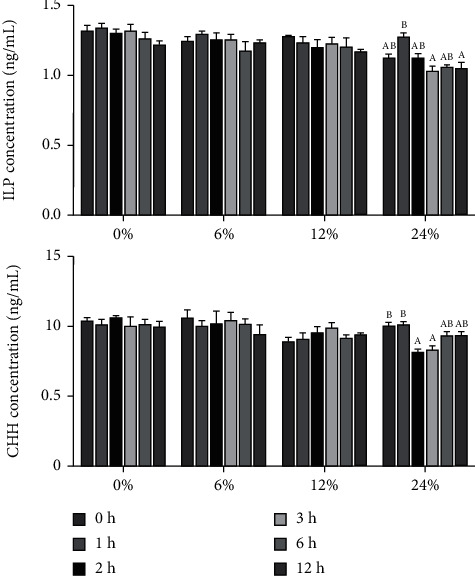
Effects of dietary corn starch levels on hemolymph hormone concentrations at different time points (0, 1, 2, 3, 6, and 12 h) in *Portunus trituberculatus*. Values are expressed as the means ± SE (*n* = 3). Means in each bar with different superscript letters are significantly different (*P* < 0.05). ILP: insulin-like peptide; CHH: crustacean hyperglycemia hormone.

The effects of different corn starch levels on hormone concentrations in hemolymph at different time points are shown in Figure 3. There were no significant differences in the insulin-like peptide (ILP) and crustacean hyperglycemic hormone (CHH) concentrations among crabs fed diets with 0, 6, and 12% corn starch (*P* > 0.05). However, in the 24% corn starch level, the highest concentration of ILP in hemolymph reached a peak at 1 hour after feeding (*P* < 0.05). The lowest CHH concentration occurred at 2 hours after feeding (*P* < 0.05).

### 3.4. Hepatopancreatic Energy Homeostasis

**Figure 4:**
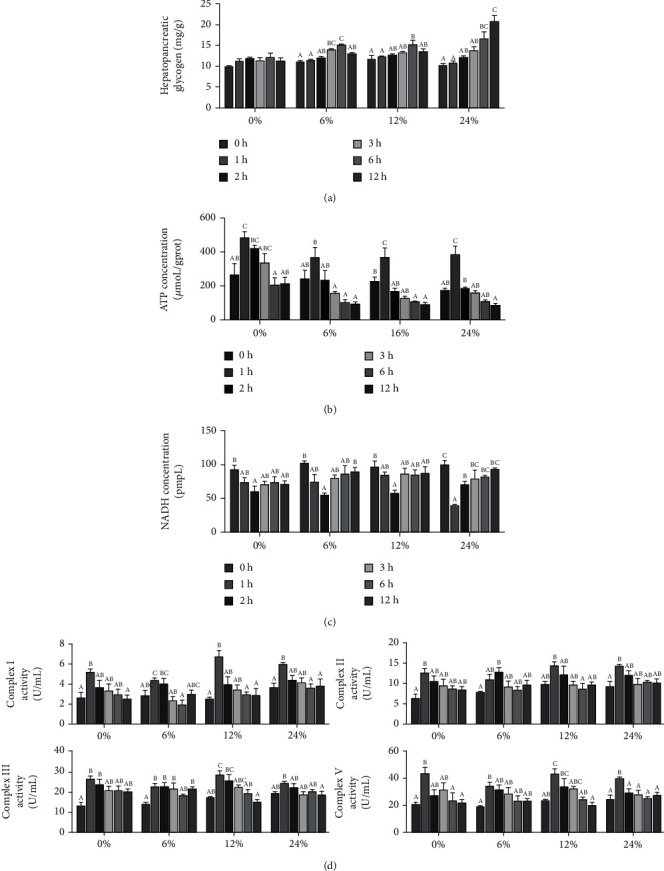
Effects of dietary corn starch levels on hepatopancreatic energy homeostasis at different time points (0, 1, 2, 3, 6, and 12 h) for *Portunus trituberculatus*. Values are expressed as the means ± SE (*n* = 3). Means in each bar with different superscript letters are significantly different (*P* < 0.05).

The effects of different corn starch levels on hepatopancreatic energy homeostasis of swimming crabs at different time points are presented in Figure 4. In the 0% corn starch level, hepatopancreatic glycogen content was not significantly influenced by dietary corn starch levels at different time points (*P* > 0.05). However, in the 6 and 12% corn starch levels, the lowest glycogen content in hepatopancreas was observed at 0 and 1 hour after feeding (*P* < 0.05), and the peak value was reached after 6 hours of feeding. However, in the 24% corn starch level, hepatopancreatic glycogen content significantly increased with time points increasing from 0 to 24 hours (*P* < 0.05) (Figure 4(a)). In 0, 6, 12, and 24% corn starch levels, the ATP content in hepatopancreas presented a trend of first increasing and then decreasing (*P* < 0.05), and the highest hepatopancreatic ATP content occurred at 1 hour after feeding (Figure 4(b)). In the 0, 6, and 12% corn starch diets, the lowest NADH content in hepatopancreas reached at 2 h of feeding; however, in 24% corn starch level, the lowest value was observed at 1 h after feeding (Figure 4(c)). In the diets with 0, 6, 12, and 24% corn starch levels, the activities of the hepatopancreatic mitochondrial respiratory chain complexes I, II, III, and V increased first and then decreased (*P* < 0.05) (Figure 4(d)).

### 3.5. Expression Levels of Genes Related to Glucose Metabolism, Transport, and Glycogen Synthesis in Hepatopancreas

**Figure 5:**
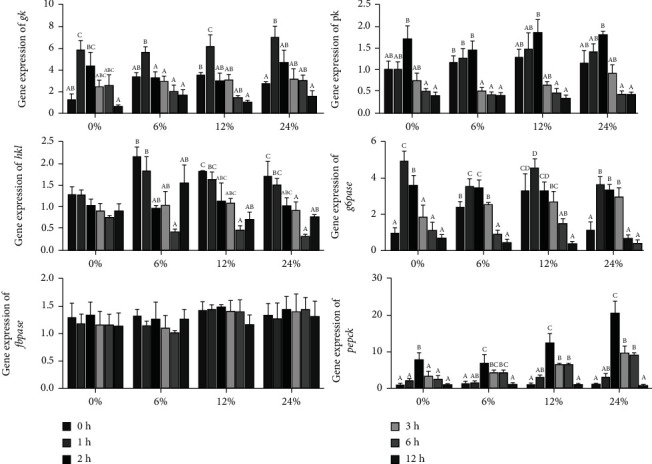
The expression levels of genes related to glycolysis and gluconeogenesis in the hepatopancreas of *Portunus trituberculatus* fed with dietary corn starch levels at different time points (0, 1, 2, 3, 6, and 12 h). Values are expressed as the means ± SE (*n* = 3). Means in each bar with different superscript letters are significantly different (*P* < 0.05). *gk*: glucokinase; *pk*: pyruvate kinase; *hk1*: hexokinase 1; *g6pase*: glucose-6-phosphatase; *fbpase*: fructose-1,6-bisphosphatase; *pepck*: phosphoenolpyruvate carboxykinase.

The effects of different corn starch levels on the expression of genes related to carbohydrate metabolism in the hepatopancreas at different time points are shown in Figure 5. In the 0, 6, 12, and 24% corn starch diets, the expression levels of *gk*, *pk*, *g6pase*, and *pepck* were significantly upregulated first and then downregulated (*P* < 0.05). There was no significant difference in *hk1* of crabs fed diet with 0% corn starch; however, in the 6, 12, and 24% corn starch diets, the expression of *hk1* was first downregulated and then upregulated (*P* < 0.05), and the lowest *hk1* was observed at 6 h after feeding. The expression of *fbpase* was not significantly influenced by dietary corn starch levels at different time points (*P* > 0.05).

**Figure 6:**
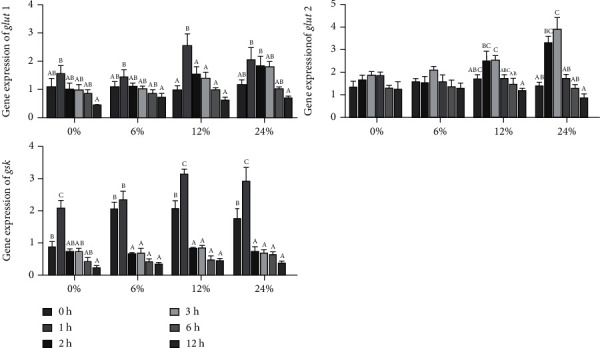
The expression levels of genes related to glucose transport and glycogen synthesis in the hepatopancreas of *Portunus trituberculatus* fed with dietary corn starch levels at different time points (0, 1, 2, 3, 6, and 12 h). Values are expressed as the means ± SE (*n* = 3). Means in each bar with different superscript letters are significantly different (*P* < 0.05). *glut1*: glucose transporter 1; *glut2*: glucose transporter 2; *gsk*: glycogen synthase kinase.

In the 0, 6, 12, and 24% corn starch diets, both *glut1* and *gsk* were significantly increased at first and then decreased significantly (*P* < 0.05, Figure 6), and the highest *glut1* and *gsk* were presented in 1 h after feeding. The expression of *glut2* of crabs fed diets with 0 and 6% corn starch was not significantly influenced in different time points (*P* > 0.05, Figure 6), and in the 12 and 24% corn starch diets, the expression of *glut2* increased first and then decreased significantly (*P* < 0.05, Figure 6), and the highest expression of *glut2* was at 2 h after feeding.

### 3.6. Expression Levels of Genes Involved into Hepatopancreas Insulin Signaling Pathway

**Figure 7:**
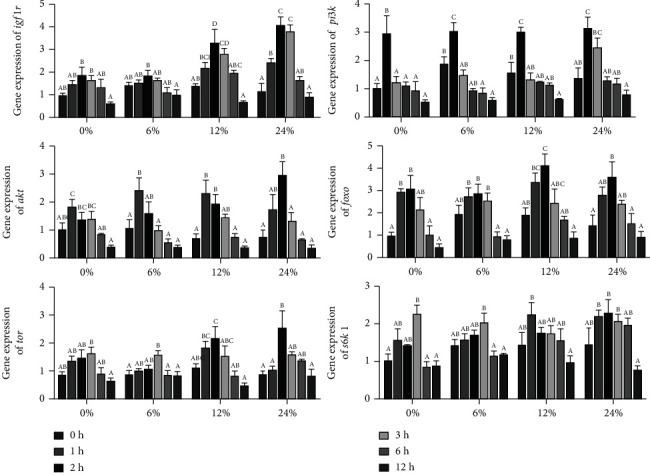
The expression levels of genes related to insulin-like metabolism in the hepatopancreas of *Portunus trituberculatus* fed with dietary corn starch levels at different time points (0, 1, 2, 3, 6, and 12 h). Values are expressed as the means ± SE (*n* = 3). Means in each bar with different superscript letters are significantly different (*P* < 0.05). *igf1r*: insulin-like growth factor 1 receptor; *pi3k*: phosphoinositide-3-kinase; *akt*: protein kinases B; *foxo*: forkhead-box class O; *tor*: target of rapamycin; *s6k1*: ribosomal protein S6 kinase1.

The effects of different corn starch levels on the expression of genes involved into the insulin signaling pathway in hepatopancreas at different time points are presented in Figure 7. In the 0, 6, 12, and 24% corn starch diets, the expression levels of *igf1r*, *pi3k*, *akt*, *foxo*, *tor*, and *s6k1* were significantly increased and then significantly decreased (*P* < 0.05). The expression level of *igf1r* and *foxo* reached the highest at 2 h after feeding, and the expression level of *pi3k* reached the highest level at 1 h after feeding and then significantly downregulated with increase of time points. Meanwhile, the expression of *akt* was the highest at 1 h in crabs fed diets with 0, 6, and 12% corn starch levels, and the highest expression of *akt* was found at 2 h after feeding in crabs fed with 24% corn starch diet. In the 6% corn starch diet, the expression of *tor* reached the highest at 3 h after feeding, and in the 12 and 24% corn starch diets, the highest expression of *tor* was at 2 h. In the 0 and 6% corn starch diets, the expression of *s6k1* exhibited the highest when ingested for 3 h, and in the 12% corn starch diet, the highest expression of *s6k1* occurred at 1 h after feeding; however, in the 24% corn starch diet, the highest expression of *s6k1* was observed at 2 h after feeding.

### 3.7. Expression Levels of Genes Related to Energy Metabolism in Hepatopancreas

**Figure 8:**
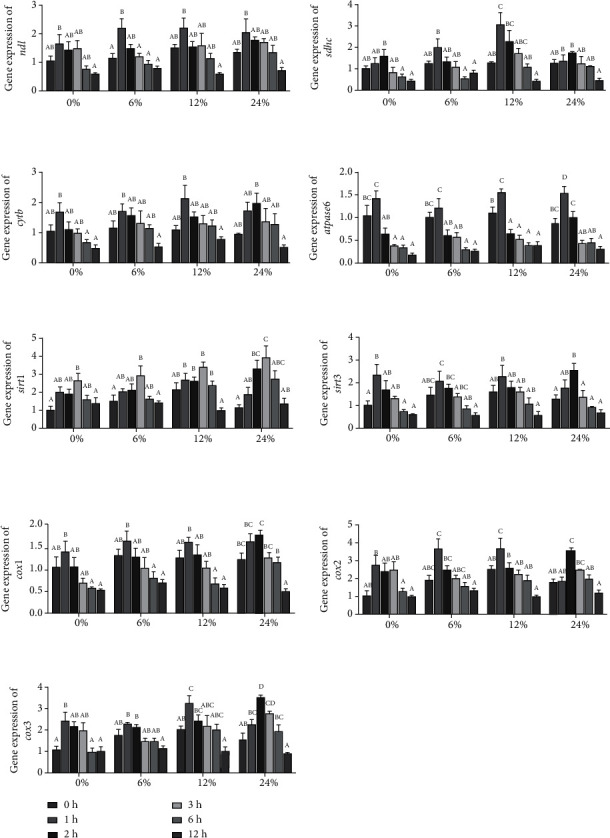
The expression levels of genes related to energy metabolism in the hepatopancreas of *Portunus trituberculatus* fed with dietary corn starch levels at different time points (0, 1, 2, 3, 6, and 12 h). Values are expressed as the means ± SE (*n* = 3). Means in each bar with different superscript letters are significantly different (*P* < 0.05). *nd1*: NADH dehydrogenase; *sdhc*: succinate dehydrogenase complex, subunit C; *cytb*: cytochrome b; *atpase6*: adenosinetriphosphatase 6; *sirt*: silent information regulator; *cox*: cytochrome coxidase.

The effects of different corn starch levels on the expression of genes related to the insulin signaling pathway in hepatopancreas at different time points are presented in Figure 8. In the 0, 6, 12, and 24% diets, the expression levels of *nd1*, *sdhc*, *cytb*, *atpase6*, *sirt1*, *sirt3*, *cox1*, *cox2*, and *cox3* all showed a trend of significant increase at first and then significant decrease (*P* < 0.05). The expressions of *nd1* and *atpase6* were the highest when ingested for 1 h, and the expression of *sirt1* was the highest when ingested for 3 h. In the 0 and 24% corn starch diets, the highest expression of *sdhc* occurred at 2 h after feeding, and in the 6 and 12% corn starch diets, the highest expression of *sdhc* occurred at 2 h. The expression of *cytb*, *sirt3*, *cox1*, *cox2*, and *cox3* in hepatopancreas of crabs fed diets with 0, 6, and 12% corn starch reached the peak at 1 h after feeding, and in 24% corn starch level, the expression level of *cytb*, *sirt3*, *cox1*, *cox2*, and *cox3* reached the highest when ingested for 2 h.

## 4. Discussion

Glucose concentrations in blood or hemolymph vary widely not only between species but also under different life histories or feeding strategies of the same breed [21]. McGarry [22] speculated that the blood glucose concentration of fish is also basically maintained at a constant level based on mammals, only fluctuating in a small range. This conclusion may not be entirely accurate. To accurately understand the tolerance of swimming crabs to carbohydrates, it is necessary to carry out glucose tolerance experiments. In the previous study, the optimal corn starch supplementation was estimated to be 8.78-9.84% for juvenile *Portunus trituberculatus* [18]. The results of present study showed that in the absence of dietary corn starch, there was no significant difference in glucose concentration of the hemolymph for swimming crabs, and a small amount of carbohydrates in other raw materials could not cause the hemolymph glucose levels of swimming crabs to change, but swimming crabs could still grow healthily. The great tolerance of swimming crabs to “low hemolymph glucose” is not possessed by mammals, which is also an interesting research direction [23]. In the 6 and 12% corn starch diets, the glucose concentration in hemolymph reached a peak after 2 hours of feeding, and the glucose concentration decreased to a minimum and remained stable after 12 hours. It was not difficult to conclude that the carbohydrate level of 6-12% was within the tolerance range of swimming crabs, and no persistent “high hemolymph glucose” phenomenon occurred. However, in the 24% carbohydrate diet, the glucose concentration reached the highest level and the “high hemolymph glucose” state lasted for 3 hours after ingestion for 3 hours, and the glucose concentration returned to the normal level after 12 hours. In the high-carbohydrate diet, the hemolymph glucose peak was delayed by 1 hour, and the hemolymph glucose level remained high. The 24% corn starch level was considered to be excessive in swimming crab commercial diet. The similar findings were frequently reported in carnivorous fish, such as flounder [10], herring (*Mylopharyngodon piceus*) [24], grouper [25], and Atlantic salmon (*Salmo salar L.*) [26]. For herbivorous aquatic animals, such as grass carp, the glucose concentration in blood peaked at 3 hours and returned to normal levels after 6 hours [24]. The blood glucose concentration of carp peaked at 3 hours and returned to normal levels after 7 hours [27].

Corresponding fluctuations in carbohydrate metabolism are accompanied by changes in hemolymph glucose levels, suggesting that glucose is an important substrate during various stimuli [28]. Glycolysis under aerobic conditions and gluconeogenesis under anaerobic conditions are important pathways of carbohydrate metabolism. Glucokinase (GK) and hexokinase (HK), phosphofructokinase (PFK), and pyruvate kinase (PK) can all limit the glycolytic pathway and affect the breakdown of glucose for energy. In this study, the enzymatic activities of PK and GK in the hemolymph showed a trend of increasing first and then decreasing. And the expression levels of genes *gk* and *pk* in the hepatopancreas also showed a similar trend, but the expression level of *hk1* was first downregulated and then upregulated. Many studies indicated that the change of glucose content is not directly related to the expression level of *hk1* [29–34]. GK and HK perform similar functions. GK is highly specific for glucose. The increase in glucose content can induce an increase in GK enzyme activity. However, HK is not specific and is inhibited by glucose-6-phosphate and ADP. This may be the reason why the expression level of *hk1* is inconsistent with the change in glucose content. Conversely, the gluconeogenesis pathway is the process of synthesizing glucose. The expression levels of genes related to gluconeogenesis such as *pepck*, *fbpase*, and *g6pase* in the hepatopancreas showed a trend of first increasing and then decreasing. Previous studies have demonstrated that the upregulation of glycolysis-related genes is often accompanied by the downregulation of gluconeogenesis-related genes [12, 28]. However, the results of this experiment showed that expression levels of gene related to glycolysis and gluconeogenesis in the hepatopancreas were consistent [18]. The correlation and difference between the carbohydrate metabolism mechanisms of crustaceans and fish remain to be explored and verified.

The balance between glucose storage and production is critical for maintaining glucose homeostasis and depends on the regulation of the activity and gene expression of key enzymes involved in glycolysis, gluconeogenesis, and glycogen synthesis and breakdown [35]. In crustaceans, glucose is primarily stored in the muscle and hepatopancreas as glycogen, and many studies have shown that excess glucose can synthesize more glycogen, which is first consumed when the body provides energy [4, 15, 36]. In the present study, there was no significant change in the hepatopancreatic glycogen concentration of crabs fed with 0% corn starch diet, which was highly consistent with the glucose content. In the 6 and 12% corn starch diets, hepatopancreatic glycogen content first increased and then decreased. And in the 24% corn starch diet, the glycogen content in the hepatopancreas was consistently increased. Obviously, swimming crabs cannot synthesize all the excess carbohydrates into glycogen in a short time. After glucose enters the body, glycogen synthase (GS) plays a key role in the process, and glycogen synthase kinase (GSK) regulates the conversion of glucose to glycogen through the phosphorylation of glycogen synthase [37]. Therefore, the expression level of *gsk* in the hepatopancreas also showed a trend of increasing first and then decreasing. However, glucose cannot enter the body directly, which is first taken up by the hepatopancreatic epithelial cells of crustaceans and then transported throughout the organism via the hemolymph [5]. Glucose also cannot pass through the lipid bilayer of the hemolymph membrane, and it must enter the cell through the family of glucose transporters (GLUTs) on the cell membrane. GLUT1 is an evolutionarily highly conserved protein responsible for glucose uptake into cells and was the first family of glucose transporters to be cloned [38]. GLUT2 plays a key role in the glucose signaling pathway for intracellular insulin secretion and biosynthesis [39]. In this study, the glucose transporter genes such as *glut1* and *glut2* were indeed activated after ingesting glucose, and both showed a trend of increasing first and then decreasing.

With the intake of carbohydrates, the glucose concentration in liver or hemolymph increases, and to maintain blood glucose balance, the related hormones that inhibit the increase of glucose will inevitably increase. On the contrary, the hormones that promote the increase of glucose will be inhibited. The effect of insulin on carbohydrate metabolism in fish has been extensively reviewed [23, 40, 41]. Insulin administration inhibits the glucose-sensing system in rainbow trout brains [42–44], and glucagon antagonizes insulin effects in fish, resulting in rapid and wide-ranging hyperglycemia [45, 46]. The types and physiological functions of endocrine hormones in crustaceans are significantly different from those in fish. The researchers found that there is a polypeptide molecule with a similar function to insulin, called insulin-like peptide [47, 48]. In an experiment on the Chinese mitten crab [49], it was proved that insulin-like peptide molecules have a hypoglycemic function, which is mainly expressed in endocrine organs such as hepatopancreas, eye stalk, and thoracic and abdominal nerve groups. Insulin-like peptides and insulin-like growth factor (IGF) polypeptides belong to the same polypeptide family. Gutiérrez et al. [50] reported that IGF may be involved in the regulation of carbohydrate metabolism in Pacific white shrimp. Unlike glucagon in higher animals, hyperglycemia hormone (CHH) is the only hormone known to induce significant upregulation of hemolymph glucose in crustaceans. Currently, hormones present in the eye stalk include hyperglycemia hormone, gonadal-stimulating hormone, gonadal-suppressing hormone, and ecdysone-suppressing hormone [49]. A variety of CHH molecules constitute the CHH molecular superfamily. Although the hyperglycemic activity of CHH has been demonstrated, the regulation of its function at the molecular level has been poorly studied [51]. In the present study, no significant differences were found in insulin-like peptides and hyperglycemic hormones at lower corn starch levels (0, 6, and 12%), but when the crabs ingested excess corn starch (24%), insulin-like levels increased markedly and regulated the stability of glucose, and hyperglycemia hormones were inhibited. After glucose levels drop, insulin-like levels drop and hyperglycemic hormones rise. At the same time, the increase of glucose concentration will also activate the insulin/IGF signaling pathway [18]. In this study, the expression level of insulin-like growth factor 1 receptor (*igf1r*) first increased and then decreased, which was highly consistent with the changes in glucose content, while phosphoinositide 3-kinase (*pi3k*), forkhead box O (*foxo*), protein kinase B (*akt*), target of rapamycin (*tor*), and ribosomal protein S6 kinase 1 (*s6k1*) all showed the same trend. The results showed that after the activation of the insulin/IGF pathway, the signal may be transmitted from the *igf1r* genes to the PI3K/AKT signaling pathway, which in turn stimulates genes *foxo* and *tor*, and the activation of the tor signaling pathway affects the expression of *s6k1*. But the specific mechanism has yet to be verified [18].

Endogenous and exogenous factors will change the balance of energy demand and supply in the body. In order to cope with the changes in the balance and maintain energy stability, the body will regulate the production of ATP by regulating the metabolic state of mitochondria. Meanwhile, mitochondrial energy metabolism may be related to *β*-oxidation [52]. Glucose can only directly generate a small amount of ATP through glycolysis, tricarboxylic acid cycle, and other pathways [28], while most ATP mainly passes through the mitochondrial *β*-oxidation system, which oxidizes the long-chain acyl-coenzyme entering the mitochondria to acetyl-CoA, and produces reducing substances (FADH_2_ and NADH), and the electrons of these reducing substances participate in the generation of ATP through the mitochondrial electron transfer respiratory chain [28, 53]. In this study, after swimming crabs ingested carbohydrates, the ATP concentration first increased and then decreased, which was consistent with the glucose content, while NADH was decomposed into ATP, and the NADH concentration first decreased and then increased, just opposite to the concentration of ATP. Similarly, the activity of mitochondrial respiratory chains I, II, III, and V, which play a key role in the process of generating ATP, also showed a trend of first increasing and then decreasing. The previous findings also demonstrated that changes in mitochondrial respiratory chain complex activity can lead to changes in tissue aerobic capacity, thereby affecting aerobic metabolism and ATP production [54]. Previous studies have reported that the expression levels of ATPase (*atpase*), NADH dehydrogenase (*nd*), succinate dehydrogenase complex subunit C (*sdhc*), cytochrome b (*cytb*), cytochrome c oxidase (*cox*), and silencing information regulator (*sirt*) were closely related to the synthesis of mitochondrial complexes [19, 55, 56], and in this study, genes related to energy metabolism all showed a trend of increasing first and then decreasing.

In conclusion, the results of the present study indicated that low carbohydrate in diet resulted that there was no significant difference in hemolymph glucose concentration and hepatopancreatic glycogen. However, dietary excessive carbohydrates (24% corn starch level) led to the “high hemolymph glucose” which lasted for 3 hours, and the glucose concentration returned to normal levels after 12 hours. At the same time, the glycogen content and insulin-like peptides of crabs fed diet with 24% corn starch significantly increased with an increase of time points after feeding. The results indicated that swimming crabs were unable to convert all glucose into glycogen in a short time and need insulin-like peptides to regulate hemolymph glucose balance. The expressions of genes related to glycolysis, gluconeogenesis, glucose transport, glycogen synthesis, insulin signaling pathway, and energy metabolism were also affected. The results also indicated that the enzymatic machinery related to glucose metabolism could regulate expeditiously to compensate the glucose load generated by feeding different carbohydrate levels for swimming crab. The tolerance to glucose is also reflected by an enhanced use of glucose through glycolysis in the hepatopancreas. Moreover, glucose loading led to a significant disturbance of glucose homeostasis which was confirmed by increased activity of insulin, glycolysis, and glycogenesis, along with gluconeogenesis suppression.

## Figures and Tables

**Table 1:** Ingredients and proximate composition of the experimental diets (%, dry matter).

Ingredients	Dietary corn starch levels (%)			
	0	6	12	24
Fish meal^a^	28.00	28.00	28.00	28.00
Shrimp meal^a^	3.00	3.00	3.00	3.00
Soybean meal^a^	15.00	15.00	15.00	15.00
Soy protein concentrate^a^	13.00	13.00	13.00	13.00
Poultry by-product meal^a^	2.00	2.00	2.00	2.00
Corn starch	0.00	6.00	12.00	24.00
Fish oil^a^	1.50	1.50	1.50	1.50
Soybean oil^a^	1.50	1.50	1.50	1.50
Soy lecithin^a^	2.00	2.00	2.00	2.00
Choline chloride	0.50	0.50	0.50	0.50
Ca(H_2_PO_4_)_2_	1.00	1.00	1.00	1.00
Vitamin mixture^a^	0.50	0.50	0.50	0.50
Mineral mixture^a^	1.00	1.00	1.00	1.00
Sodium alginate	1.00	1.00	1.00	1.00
Cellulose	30.00	24.00	18.00	6.00
Proximate composition (%)				
Moisture	10.11	10.46	10.04	10.90
Crude protein	42.83	43.01	43.14	43.12
Crude lipid	6.27	6.65	6.03	6.33
Ash	8.12	7.83	8.16	7.64
Crude fiber	30.53	24.62	18.63	6.71
NFE^b^	2.14	7.43	14.00	25.30
Gross energy (kJ/g)^c^	12.95	14.06	14.97	16.91

^a^All raw materials are purchased from TeckBank Co., Ltd. ^b^Nitrogen − free extract = 100% − (moisture% + crude protein% + crude lipid% + ash% + crude fiber%). ^c^Energy = (crude protein% × 23.6 + crude lipid% × 39.50 + starch% × 17.2)/100.

**Table 2:** Real-time PCR primer sequences of gene expression in hepatopancreas of swimming crab.

Gene names	Primers		Amplification efficiency	Product size	Access no.
	Forward (5′-3′)	Reverse (5′–3′)			
*gk*	AGGTGGACCAACACTCTCGC	TCCAGCAAGCCACAGGTCTC	98.4	184	XP_023223115
*pk*	ACACCAAAGGACCCGAAATC	ATGCCGCCATTCTCTACCTC	99.1	268	ALK82311
*hk*	TATGTGGCAGGAATCGTGTC	GGAGTCTATCAGCAATGGCG	97.4	256	ABO21409
*g6pase*	ATGTGGACGCTGCTCTTCTG	CACCATCCAAGTGGCATACC	93.1	273	ALK82315
*fbpase*	CAGAAGAAAATCCCTCACGC	CCACATACTTCCCCTGACGA	97.2	286	AMJ52089
*pepck*	CGAGCCGCTACCCAAATA	CGAAGTCGTCCTCGTTGA	93.7	290	AAL78163
*glut1*	TGGTGCGGAACTCCAATCTA	GAAGCCTATGCCGACAATGA	92.4	296	AIT97017
*glut2*	CGATGGGAGCCTTGAGTTTT	ACAGGATTCCAACCACGACC	94.1	312	ALG65274
*gsk*	AGGTGCTCCAGGATAAACGG	ACACACCCAGCGAGTGAATG	96.4	276	ASW35107
*foxo*	CATAAGTTCTCGCCAGCCTC	TAACCTTCAGGACACGGGAG	92.3	108	XP_023216972
*igf1r*	CACTCACCAGGAGCCCATCTA	TCAGAGAGTTTCACAACCCGC	98.6	261	XP_023716772
*pi3k*	AGCCACCACTCGCTGAACA	GGGATGGGACTCTGCTGAAG	95.3	124	ADE44090
*nd1*	CGAAGCCGAGGTAGTGTA	CGATTTTGCTGAAGGAGA	91.3	228	Zhao et al. [55]
*sdhc*	CGGCTCCTACCCACACTACT	CCCAAATCCCACACCAAG	97.1	179	Zhao et al. [55]
*cytb*	GAACTACGGTTGACTTCTACG	AGTAATAACTGTTGCCCCTC	91.8	223	Zhao et al. [55]
*Atpase6*	TAGCACTCTCTCTACCTTT	AGCAAGTGTTCCTGGTC	94.3	167	Zhao et al. [55]
*cox1*	TATTGTAAGTCAAGAGTCCG	CTCACAGCATAGAAGGTC	93.4	254	Zhao et al. [55]
*cox2*	GTGAATAACCCGTCTGTAACTT	CATCTAATAATCGGAACCCTG	94.8	145	Zhao et al. [55]
*cox3*	CAAAGGTTTACGGTGAGGT	CCATAGGGAGGTCAGTTCAT	92.7	132	Zhao et al. [55]
*sirt1*	CTCCACCACTTCCAACCTTAG	CCAGCACCAGTCAATACGATG	100.6	318	Zhao et al. [55]
*sirt3*	CAACACTGCTCACCACTTCC	TGGCTTCACTTTGCCCTTA	96.6	259	Zhao et al. [55]
*tor*	TGTGGACATAGGGCAAACTG	GACCGCTTCACCAAATCATC	88.24	174	Wang et al. [57]
*s6k1*	CGCCCCTCAGATTTCCAGT	TCTCAGCCTTTGTGTGCG	102.65	179	Wang et al. [57]
*akt*	GGACTACGAGGCACCAAGAA	TGGACCACTTCATCACGCTC	87.92	175	Wang et al. [57]
*β-actin*	GAAGTAGCCGCCCTGGTTGT	GAATACCTCGCTTGCTCTGC	102.6	—	Pan et al. [58]

## Data Availability

The data used to support the findings of this study are included within the article.
